# Disinvestment initiatives in Malaysia healthcare system: the journey from possibility to reality

**DOI:** 10.3205/hta000140

**Published:** 2024-11-19

**Authors:** Hanin Farhana Kamaruzaman, Ku Nurhasni Ku Abd Rahim, Mudla Mohamed Ghazali Izzuna

**Affiliations:** 1 Malaysian Health Technology Assessment Section (MaHTAS), Medical Development Division, Ministry of Health, Putrajaya, Malaysia; 2 Health Economics and Health Technology Assessment (HEHTA), School of Health and Wellbeing, University of Glasgow, UK

**Keywords:** disinvestment, health technology reassessment, low-value care, health technology management, benefits package

## Abstract

Disinvestment in healthcare allows for strategic reallocation of resources from low-value care to higher-value areas, particularly in promoting clinical effectiveness, improving patient outcomes, and long-term cost savings. The Malaysian Health Technology Assessment Section (MaHTAS) is investigating the incorporation of a disinvestment framework into the health technology life cycle, in accordance with the Ministry of Health Malaysia's recent healthcare transformation strategy. Several health technology assessment (HTA) reports by MaHTAS have integrated concepts of health technology reassessment, with an emphasis on effectiveness and adverse effects. However, the need for additional funds and implementation strategies has impeded the impactful and timely execution of HTA recommendations. This article highlights ongoing efforts to promote disinvestment activities in the Malaysian healthcare system by raising early awareness and engaging with healthcare stakeholders during the planning phase. The journey from the possibility to the reality of implementing healthcare disinvestment initiatives in Malaysia requires addressing both facilitators and challenges. Facilitators identified include strong political will and the presence of global support for collaboration and knowledge sharing, among others. Some of the recognized challenges are getting the trust of the stakeholders, the need for additional funding to support disinvestment programs, and the lack of expertise as well as guideline to carry out the disinvestment process. By highlighting the steps taken and the strategic planning required, this article sheds light on the potential for disinvestment to enhance the efficiency and effectiveness of healthcare delivery in Malaysia, ultimately contributing to a more sustainable healthcare system.

## Introduction

Resource allocation is essential in the healthcare sector for defining the quality and efficiency of services. Resources are frequently dispersed unevenly, with a notable fraction allocated to low-value care at the expense of neglecting higher value technology. This misallocation can lead to insufficient results for patients and the healthcare system overall. Disinvestment can effectively reallocate resources from low-value care to better value technologies to address this issue [1]. It is described as the process of shifting resources away from programs or interventions that are deemed ineffective or of little value, towards those with more potential to enhance patient outcomes and provide better value for healthcare spending [2]. By implementing disinvestment strategies, healthcare organizations can redirect financial resources towards investments in cutting-edge technologies and innovative treatments that offer greater benefits to patients.

Establishing a connection between health technology assessment (HTA) and reimbursement decisions offers a powerful incentive for the identification and evaluation of potentially beneficial new technologies. In addition to the fact that there are disincentives for disinvestment, what is more complicated is implementing the strategy to identify the candidates that are considered low-value technologies [3]. The available frameworks are mainly applicable to the specific healthcare system, which could have different health financing mechanisms and resource allocation processes from one to another. However, it is also worth noting that rationing, disinvestment, and health technology reassessment do not occur in a vacuum, nor do reimbursement decisions. Those engaging in reimbursement decisions and disinvestment are constantly aware of the costs, even if cost reduction or capital reallocation is not their primary purpose [4]. 

**Figure 1:**
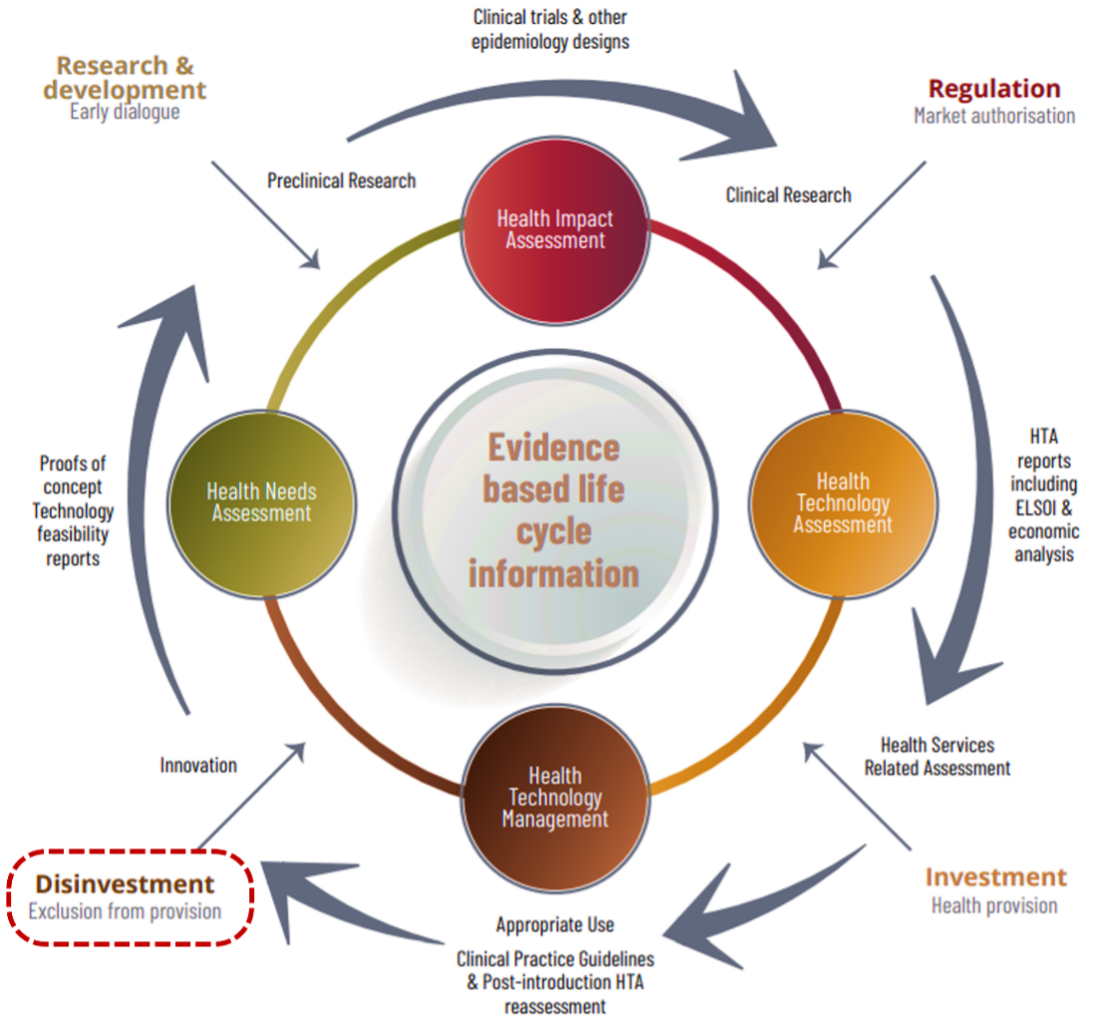
Life cycle approach of health technologies using HTA and Horizon Scanning in Malaysian healthcare system [22]

Another strong link in relation to disinvestment program is the existence of at least an HTA agency in that country [5]. The Malaysian Health Technology Assessment Section or known as MaHTAS is the single HTA agency in Malaysia and was established almost three decades ago in 1995. The key catalyst of the initial establishment of this section was the urgent need for a robust mechanism to prioritize and select new health technologies for integration into the healthcare system [6]. This was done in response to the growing demand for the newest and sophisticated technologies among the public and healthcare professionals with the ultimate objective of improving the standard of care. In line with the recent healthcare transformation plan within Ministry of Health Malaysia, MaHTAS is investigating the possibility of incorporating disinvestment framework as part of the life cycle approach of HTA process (Figure 1 (Fig. 1)). However, it is noteworthy that the leading force is not merely to close this loop in the technology life cycle. Rather, the focus is on supporting resource allocation decisions and health technology management based on scientific evidence.

The planning of disinvestment activities by MaHTAS began in 2019, with a proactive approach to identifying potential implementation strategies. A series of workshops were held as part of the World Health Organization’s (WHO) consultation program, with the goal of systematically identifying candidates for health technology reassessments. These workshops facilitated multi-stakeholder involvement by using the collective expertise of healthcare professionals, regulatory bodies, research institutes, and international networks with experience in disinvestment programs such as EuroScan. The discussions focused on the sources of identification for health technologies that were deemed to be of no or low benefit, as well as developing the prioritization criteria for evaluating candidates for disinvestment. The provisional decision from these workshops was to use comparable priority criteria that echoed the concepts of the Pritec Tool, including population impact, risk-benefit analysis, financial implications, and organizational feasibility [7]. This approach demonstrated MaHTAS' dedication to using HTA as a tool for reassessing low-value care and health technologies within the Malaysian healthcare system.

In this article, we will outline the continuing efforts in developing a disinvestment framework and reassessment of health technologies in Malaysia, with a particular emphasis on the activities carried out to prepare for its implementation in the near future. In addition, this study will highlight the facilitators and challenges reported by healthcare stakeholders, as well as recommendations for future works in the context of disinvestment initiatives in Malaysia.

## Shifting from a low-value care to a higher value technology

The concept of disinvestment in healthcare offers a strategic avenue for reallocating resources from low-value care to higher value areas, particularly in technological improvement. By divesting from practices, treatments, or technologies that yield marginally low benefits compared to their costs, healthcare systems can redirect these resources towards innovative technologies and interventions with greater clinical effectiveness, better patient outcomes, and long-term cost savings. 

Within the Malaysian healthcare setting, transitioning from traditional Pap smear to HPV DNA-based cervical cancer screening reflects a deliberate shift from low-value to higher-value strategy by more efficiently allocating healthcare resources. An HTA report by MaHTAS published in 2011 partially influenced this decision, suggesting that the cervical cancer screening program should incorporate HPV DNA-based testing as a primary screening strategy [8]. HPV DNA screening is more sensitive and specific than Pap smears, enabling the earlier and more precise diagnosis of high-risk HPV strains that cause cervical cancer. This move can considerably minimize the incidence of false negatives and needless follow-up treatments, thereby reducing the overall load on healthcare systems.

This strategy also encourages the increased utilization of evidence-based technology by directing resources towards interventions that yield the highest benefits. The HTA report recommended performing HPV DNA-based testing every five years due to the test's high cost at the time. Finally, this strategy not only enhances the quality of cervical cancer screening, but it also adheres to the principles of HTA by emphasizing high-value treatment and maximizing health benefits for the community. The implementation of Program ROSE (Removing Obstacles to Cervical Screening) exists within the larger Action Plan Towards Elimination of Cervical Cancer in Malaysia (2021–2030) which encompasses elimination goals and targets for the scale-up of vaccination, cervical cancer screening and treatment [9]. It took nearly a decade from HTA recommendation in 2011 to policy approval in 2020, which demonstrates the resource-intensive and time-consuming nature of HTA implementation. Additionally, it also emphasizes the need to assess whether HTA alone is sufficient to drive disinvestment or if complementary strategies are required.

Another important trigger is the safety or adverse effects of a particular health technology. An HTA by MaHTAS on intraocular lens (IOL) could reflect the possibility of disinvestment recommendations based on the safety issues of the current technology being practiced. Reports of late postoperative opacification of IOL particularly with certain hydrophilic acrylic designs, which necessitated explantation due to dystrophic calcification, prompted an assessment comparing the safety of hydrophilic acrylic and hydrophobic IOL implants for adult cataract surgery in Ministry of Health facilities [10]. The review found a weak to fair level of evidence suggesting that opacification affecting vision was primarily associated with hydrophilic acrylic IOLs rather than hydrophobic acrylic ones, attributed to calcium and phosphate deposition on the surface or within the optic material. However, the exact pathophysiology of these complications remains incompletely understood, with diabetic patients being more frequently and severely affected. Patients who have received hydrophilic acrylic IOLs require longer and more frequent follow-up, particularly if they have risk factors such as diabetes. As a result, the HTA report recommends using hydrophobic acrylic IOLs for cataract surgery in Malaysia and proposed an incident reporting approach for IOL opacification, irrespective of material or design, to enhance local comprehension of the issue.

## Early awareness on disinvestment and engagement with healthcare stakeholders

In fostering discussions on disinvestment among key Malaysian healthcare stakeholders, a pivotal workshop was organized by MaHTAS in June 2022 with the primary aim of introducing the concept of disinvestment and exploring the feasibility of incorporating value assessment into the prioritization of resource allocation decision-making at the hospital level. Attended by directors from both specialist and non-specialist public hospitals, the event provided a crucial platform for these leaders to engage with the idea of optimizing healthcare resources.

**Figure 2:**
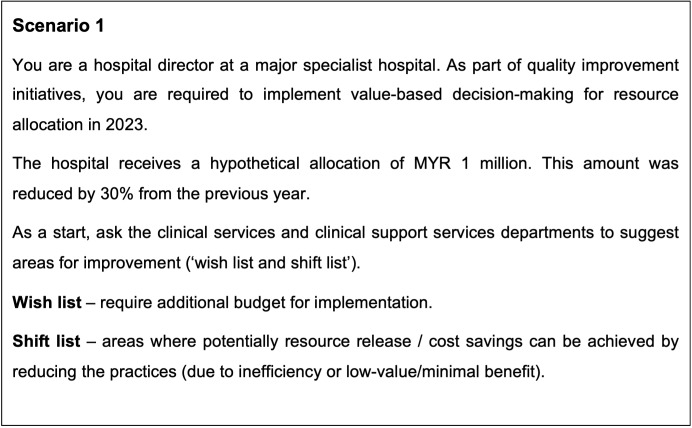
Value based decision-making scenario (For this exercise, both hypothetical lists were as described in Attachment 1)

At the outset, participants were introduced to the considerations for investment and disinvestment decision-making, as well as the Programme Budgeting and Marginal Analysis (PBMA) method for disinvestment. They were then divided into five groups and tasked with executing a value-based decision-making exercise detailed in Figure 2 (Fig. 2) (for this exercise, both hypothetical lists were as described in Attachment 1), which was to prioritize programs for additional funding while identifying low-value existing services from which resources could be released. By focusing on the value of disinvestment, the workshop sought to equip hospital directors with the knowledge and tools needed to make informed decisions that could enhance the efficiency and effectiveness of healthcare delivery. This initiative marks an important step towards integrating value-based decision-making in Malaysian public hospitals, with the potential to significantly improve patient outcomes and resource utilization.

The workshop focused on identifying investment and disinvestment criteria, ways to provide evidence of efectiveness, information on expenditure, potential risks, and impacts. Participants presented their selection of interventions for both lists and deliberated with expert panels. While all groups used similar criteria for prioritization and decision-making, such as clinical effectiveness, safety, disease burden, suitability, benefit receiver, available alternatives, potential risk, human resources, and economic considerations; they made different decisions on resource allocation. However, non-clinical interventions like hospital catering services were unanimously disinvested. The results were summarized in Attachment 2.

The deliberative discussion highlighted several challenges in resource allocation at the hospital level. Firstly, investment and disinvestment of resources will need buy-in from healthcare providers who have experience in practice. Their active participation in decision-making will have a huge impact on managing expectations when there are changes in practice resulting from resource reallocation. Moreover, the shifting of resources may need to consider the magnitude of the impact on the services, as the hospital implementing the changes may also be responsible for other smaller hospitals, for instance, in a hospital cluster system. Furthermore, new investments may demand additional resources for implementation; hence, financial planning for new interventions or programs is required. The discussions also emphasized the crucial role of a monitoring and auditing framework in supporting resource allocation decisions, particularly during the transition from existing to new services.

From this workshop, we concluded that the concepts of disinvestment and value assessment in prioritizing resource allocation have received positive feedback from these key healthcare stakeholders. We also discovered that a structured method, such as PBMA, is an effective instrument for resource reallocation and that stakeholder engagement is critical for a successful prioritizing process and decision-making. Nonetheless, disinvestment should not compromise access to services or quality of care in order to prevent equity concerns and rejection of disinvestment decisions.

Building on previous stakeholder engagement, mixed-method research was carried out to address the major concerns of healthcare disinvestment in Malaysia, which included an online survey and in-depth interviews with key stakeholders. This study is part of a doctoral research project at the University of Glasgow and is currently a work in progress. The online survey revealed that although Malaysian healthcare views disinvestment as a priority for effective resource allocation, it lacks a systematic structure for its implementation. The majority (90%) of the respondents agreed that a formal disinvestment process is required and training is necessary to ensure the success of the program. 

We identified clinical efficacy and cost-effectiveness as the most critical factors for disinvestment. Although equity was regarded as the least important factor, the most pressing issue is access to treatment and availability of necessary care, particularly for underprivileged or vulnerable people. According to the study, main challenges to disinvestment adoption include insufficient stakeholder support, political will, and a lack of expertise in carrying out the process. These findings are consistent with previous studies that highlighted several main barriers to the implementation of disinvestment decisions, namely stakeholder resistance to change practices [11], [12], insufficient support and leadership from those with authority [13], and a lack of guidance and skilled experts in the disinvestment process [14].

## Support, knowledge sharing and increasing interest from global platforms

Global networks play an important role in facilitating healthcare disinvestment by encouraging support and information exchange. Several significant programs demonstrate this collaborative approach, such as the Disinvestment and Early Awareness Interest Group (DEA-IG) that has been established by the Health Technology Assessment international (HTAi) to address challenges associated with healthcare disinvestment [15]. This group focuses on creating platforms for formal discussion on disinvestment in HTAi, as well as proposing methodological frameworks and guidelines that can assist healthcare organizations in identifying and discontinuing low-value care and interventions. The HTAi DEA-IG promotes international collaboration, enabling the exchange of best practices and methodologies, thereby supporting more informed and effective disinvestment decisions globally.

The international Health TechScan (i-HTS) is also among the key players in the global effort to support disinvestment. Formerly known as EuroScan, i-HTS promotes the use of HTA for evaluating and optimizing healthcare technologies, thereby ensuring efficient utilization of resources. Comprising of policymakers, experts, and practitioners from various countries, i-HTS facilitates the sharing of experiences and strategies related to health system improvements, including the discontinuation of ineffective interventions [16]. As an active member of both HTAi and i-HTS, MaHTAS collaborated closely with these groups on the early awareness alert system and horizon scanning. In the future, we are looking forward to working in concert with DEA-IG and i-HTS to ensure continuous effort in sharing best practices in healthcare disinvestment and health technology reassessment.

Furthermore, the COVID-19 epidemic has intensified the emphasis on healthcare resource allocation, resulting in a greater interest in studies related to the methodological approach to disinvestment and reassessment of low-value care [4]. This rise in academic attention emphasizes the importance of evidence-based decision-making in improving healthcare system resilience and responsiveness. Even before the global health tragedy, researchers are exploring the impacts of resource constraints and the urgent need for active identification of no- or low-added value technologies, which has increased the necessity of effective disinvestment strategies [17]. As a result, there is a tendency to combine priority setting and resource allocation for investment in high-value care with opportunities to disinvest in low-value care and obsolete technologies.

## Challenges in implementation of healthcare disinvestmenrt in Malaysia

Implementing disinvestment initiatives in Malaysia’s healthcare system is not without hurdles. From the key informant interviews with Malaysian healthcare stakeholders, we identified several striking concerns and challenges in the implementation of disinvestment programs. One of the most significant setbacks is obtaining buy-in and support from multiple stakeholders, which is critical to the success of any disinvestment program. This process necessitates acquiring political will and sustaining motivation among all parties involved, which may be achieved through proper incentives [18]. Healthcare administrators, key leaders, and policymakers must skillfully navigate the intricate terrain of interests and priorities, guaranteeing that every stakeholder perceives their concerns and contributions as significant. Without this critical support, efforts to streamline and optimize resource allocation may face resistance or apathy, undermining the initiative's objectives.

Another key barrier is establishing mutual trust and respect among decision-makers and implementers, such as hospital managers and clinical care providers. A lack of alignment and coordination among these groups might pose substantial challenges to effective implementation. In addressing this issue, Patey et al. [19] suggested incorporating the behavior substitution component into the change management strategy, which may increase the likelihood that this technique is efficient in reducing low-value care. Such a framework may ensure that choices are made in a transparent, comprehensive, and inclusive manner, considering the perspectives and expertise of all relevant parties. This approach not only builds trust but also fosters a collaborative atmosphere and augments the chances of decision acceptance and effective action.

Furthermore, the skills, capability, and expertise to undertake and implement disinvestment recommendations at all levels – national, local, and institutional – are critical to success. It requires extensive capacity development and knowledge transfer initiatives to equip healthcare professionals with the essential skills and understanding. Moreover, the early phases of disinvestment may necessitate additional resources and funding to support these capacity-building activities and manage the transition period. Ensuring that healthcare systems are adequately prepared to implement new recommended practices and policies is vital to the long-term success of disinvestment initiatives. While budget reductions and program suspensions are common in public health service initiatives [20], financial strategies for sustainable in-house services must also be factored in.

## Recommendations and future planning

To prepare for the implementation of disinvestment initiatives within the Malaysian healthcare system, we recommend several measures and outline future planning that is currently in the pipeline for MaHTAS. A fundamental step in this process is to establish evidence-based policies for resource allocation and health financing mechanisms that are consistent with the Malaysian Health White Paper. This serves as a road map for strengthening health system governance through better regulatory frameworks, enhanced accountability, and improved policy-making processes [21]. By putting these policies in place, decision-makers may guarantee that resources are allocated to the most effective and required health technologies, thereby improving the overall quality of care while being financially sustainable. These strategies may provide a structured approach to identifying and discontinuing low-value interventions as well as reallocating funding to areas with higher health benefits.

MaHTAS is now engaged in the development of a benefits package as part of the healthcare financial sustainability goals outlined in the Malaysian Health White Paper. This is another crucial aspect that underscores the need for a comprehensive framework for the reassessment of low-value treatments and interventions. A clearly defined benefits package facilitates a methodical assessment of health technologies, guaranteeing the retention of only those that offer substantial value to patients. This framework should be versatile enough to reassess technologies periodically and timely, allowing for the identification and phasing out of low-value technologies without jeopardizing access to treatments.

Another factor that may determine the successful execution of disinvestment efforts is the customization of the process to suit local practices. Decisions to discontinue investment in low-value technology must consider the unique demands and circumstances of different healthcare settings at various levels of care provision. What is considered superfluous or low-value in one setting might be critical in another, owing to differences in facility readiness and population requirements. This calls for a nuanced approach that avoids one-size-fits-all recommendations, thus minimizing the risk of conflicting or ineffective implementations. Although PBMA and HTA have been shown to be the most used tools for disinvestment [4], we contend that these two approaches have inherent limitations in evaluating the consequences of decision-making on the de-implementation of low-value care. Therefore, we envisage that there is a more thorough methodological framework beyond HTA and PBMA that might be acceptable for implementation in Malaysia by taking into account the perspectives of local healthcare stakeholders. As previously stated, this discovery is the result of a doctoral research project involving MaHTAS researchers, which has the potential to contribute to the development of the Malaysian methodological framework for disinvestment in low-value care and health technology.

## Conclusion

In line with the recent healthcare transformation plan in Malaysia, MaHTAS is investigating the possibility of incorporating a disinvestment framework within the scope of the life cycle approach in the HTA process. This ongoing effort was started in 2019 and is currently being carried out as part of a research project by engaging Malaysian healthcare stakeholders as important key players in the implementation of disinvestment processes and decisions. We incorporate them early in the planning phase as multidisciplinary stakeholder involvement at various levels of care and governance is among the most essential factors in determining the acceptance and success of any disinvestment program.

There are previous Malaysian HTA reports that may have partially adopted the principles of health technology reassessment; however, the application of value-based decision-making is not well established in these examples. Since there is limited awareness and expertise in carrying out this initiative, active collaboration on global platforms is instrumental in advancing healthcare disinvestment by promoting knowledge sharing and exchanging views on challenges, as well as capacity building. In addressing the need for guidance on disinvestment locally, a comprehensive methodological framework beyond HTA and PBMA should be developed that builds upon the perspectives of Malaysian healthcare stakeholders.

## Notes

### Competing interests

The authors declare that they have no competing interests.

### Funding

The funding for the article preparation and processing fee is supported by the EuroScan International Network. Otherwise, this research received no specific grants from any agency, commercial or not-for-profit sectors. 

### Acknowledgments 

The authors would like to thank Dr. Hans-Peter Dauben and Maximilian Otte for the initial idea of this article. Special gratitude to the MaHTAS staff (Dr. Mohamed Hirman Hj. Abdullah, Dr. Roza Sarimin, Dr. Syaqirah Akmal, Mr. Lee Sit Wai, Dr. Sze-Shir Foo) for providing input and coordinated the Hospital Director Summit 2022 workshop on healthcare value-based decision-making, as well as all the participants involved with the exercise. We are especially indebted to Professor Olivia Wu and Dr. Eleanor Grieve for supervising Hanin Kamaruzaman’s doctoral research project on healthcare disinvestment initiatives at Health Economics and Health Technology Assessment (HEHTA), University of Glasgow. We also thank the Director General of Health Malaysia for the permission to publish this article.

### Authors’ contribution statement

We, the authors listed above, attest that

1. each author contributed to the conception, design and interpretation of information, and the writing of this paper;
2. each has reviewed and approved the version being submitted; and
3. the content has not been published nor is being considered for publication elsewhere.

### Authors’ ORCID

- Hanin Farhana Kamaruzaman:
https://orcid.org/0000-0002-4926-5232
- Ku Nurhasni Ku Abd Rahim:
https://orcid.org/0000-0003-1603-3758
- Izzuna Mudla Mohamed Ghazali:
https://orcid.org/0000-0003-1830-0485

## Supplementary Material

Hypothetical case studies for group exercise

Results from scenario analyses in group exercise
