# Supplementary material for: Disinvestment initiatives in Malaysia healthcare system: the journey from possibility to reality
Source: GMS Health Innov Technol. 2024 Nov 19;18:Doc03. doi: 10.3205/hta000140 (PMC11641420; doi:10.3205/hta000140)
Supplement: Hypothetical case studies for group exercise [file HInT-18-03-s-001.pdf]

Attachment 1

Hypothetical case studies for group exercise

Wish List

No.	Interventions	Total cost
1.	Medicine Waste Management	MYR 220,000
2.	Pulmonary rehabilitation	MYR 320,000
3.	Major system changes in acute stroke services	MYR 1,500,000
4.	Proton beam therapy for head and neck cancer in adults (comparator: Intensity modulated radiotherapy)	MYR 950,000

Shift List

No.	Interventions	Total cost
1.	Use of mucolytics in treatment of Chronic obstructive pulmonary disease (COPD)	MYR 220,000
2.	High-cost antibiotics as first line treatment	MYR 820,000
3.	Hospital catering services (reduce meal frequency)	MYR 1,000,000
4.	Red blood cell transfusion practice in intensive care unit	MYR 140,000
5.	Routine sampling of nasal polyps and tonsil post-removal for pathology/histopathology examination	MYR 26,500
6.	Serum cobalamin test in adult with new episode of unexplained fatigue	MYR 13,000

Attachment to: Kamaruzaman HF, Ku Abd Rahim KN, Izzuna MMG. Disinvestment initiatives in Malaysia healthcare system: the journey from possibility to reality. GMS Health Innov Technol. 2024;18:Doc03. DOI: 10.3205/hta000140
