# Supplementary material for: Disinvestment initiatives in Malaysia healthcare system: the journey from possibility to reality
Source: GMS Health Innov Technol. 2024 Nov 19;18:Doc03. doi: 10.3205/hta000140 (PMC11641420; doi:10.3205/hta000140)
Supplement: Results from scenario analyses in group exercise [file HInT-18-03-s-002.pdf]

Attachment 2

Results from scenario analyses in group exercise

Decision by group	Group 1	Group 2	Group 3	Group 4	Group 5
**Shift**	All interventions	1) Use of mucolytics in treatment of COPD<br>2) High-cost antibiotics<br>3) Hospital catering services	Hospital catering services	1) Use of mucolytics in treatment of COPD<br>2) Hospital catering services<br>3) RBC transfusion practice in ICU<br>4) Pathology / histopathology	1) Use of mucolytics in treatment of COPD<br>2) Hospital catering services<br>3) Serum cobalamin test
**Wish**	All interventions and re-invest a proportion in high-cost antibiotic	1) Medicine Waste Management<br>2) Pulmonary rehabilitation<br>3) Major system changes in acute stroke services	Proton Beam Therapy for head and neck cancer in adults + training	All accept Proton Beam Therapy for head and neck cancer in adults	1) Medicine Waste Management<br>2) Pulmonary rehabilitation<br>3) Major system changes in acute stroke services
**Savings from shifting resources**	MYR 2,180,000	MYR 2,000,000	MYR 1,000,000	MYR 1,500,000	MYR 1,200,000
**Investment on wish list**	MYR 3,000,000	MYR 920,000	MYR 2,000,000	MYR 1,400,000	MYR 920,000

**COPD = Chronic obstructive pulmonary disease; ICU = intensive care unit; RBC = red blood cell*

Attachment to: Kamaruzaman HF, Ku Abd Rahim KN, Izzuna MMG. Disinvestment initiatives in Malaysia healthcare system: the journey from possibility to reality. GMS Health Innov Technol. 2024;18:Doc03. DOI: 10.3205/hta000140
